# Vitamin D Beliefs and Associations with Sunburns, Sun Exposure, and Sun Protection

**DOI:** 10.3390/ijerph9072386

**Published:** 2012-07-04

**Authors:** Bang Hyun Kim, Karen Glanz, Eric J. Nehl

**Affiliations:** 1 Center for Health Behavior Research, Perelman School of Medicine, University of Pennsylvania, 110 Blockley Hall, 423 Guardian Ave., University of Pennsylvania, Philadelphia, PA 19104, USA; Email: kglanz@upenn.edu; 2 Emory Rollins School of Public Health, Emory University, 1518 Clifton Road NE, Atlanta, GA 30222, USA; Email: enehl@emory.edu

**Keywords:** sun exposure habits, vitamin D knowledge, sunscreen use, sun protection behavior

## Abstract

The main objective of this study was to examine certain beliefs about vitamin D and associations with sun exposure, sun protection behaviors, and sunburns. A total of 3,922 lifeguards, pool managers, and parents completed a survey in 2006 about beliefs regarding vitamin D and sun-related behaviors. Multivariate ordinal regression analyses and linear regression analysis were used to examine associations of beliefs and other variables. Results revealed that Non-Caucasian lifeguards and pool managers were less likely to agree that they needed to go out in the sun to get enough vitamin D. Lifeguards and parents who were non-Caucasian were less likely to report that sunlight helped the body to produce vitamin D. A stronger belief about the need to go out in the sun to get enough vitamin D predicted more sun exposure for lifeguards. For parents, a stronger belief that they can get enough vitamin D from foods predicted greater sun protection and a stronger belief that sunlight helps the body produce vitamin D predicted lower sun exposure. This study provides information regarding vitamin D beliefs and their association with certain sun related behaviors across different demographic groups that can inform education efforts about vitamin D and sun protection.

## 1. Introduction

The relationship between sun exposure and vitamin D levels has been an important topic in recent medical and community related research [1]. In the past, vitamin D was simply considered as a supplement, but recent developments show that it may have potential in preventing certain chronic diseases and improving health outcomes [1,2,3]. 

The typical recommendation for “modest” UV exposure is 5–30 minutes exposure to legs and arms three times a week, depending on skin type, location, and season [4]. Recently revised recommended dietary allowances (RDA) for vitamin D recommends serum 25-hydroxyvitamin D (25(OH)D) at or above 75 nmol/L (30 ng/mL) to sustain bone density, calcium absorption, and to minimize risk of osteomalacia and rickets [5,6,7,8,9,10,11]. The amount of UV exposure required to maintain adequate levels of vitamin D also depends on several other factors such as skin type, geographical location, occupation, sun exposure, and environmental conditions [9,12,13,14]. 

Increasing evidence of vitamin D deficiency in the general population has led to debates on the importance of sun exposure in meeting daily vitamin D requirements [1,12,15,16,17]. Furthermore, evidence of the association of skin cancer with vitamin D levels have been inconsistent [12,13,16,17,18]. Due to inconsistencies in vitamin D recommendations and competing messages, studies in Australia have shown low awareness of vitamin D and its effects [19,20]. In a recent study, many people were aware of vitamin D health benefits but 80% of people could not name one health benefit [19]. In another study with office workers, there was little knowledge of which foods were good sources of vitamin D or that sun exposure leads to the formation of vitamin D [20]. With the current limitations in knowledge and beliefs about vitamin D, it is difficult to balance a precautionary approach about the benefits of sun exposure with messages about the harms of overexposure to the sun [20]. 

Based upon the current scientific and media interest in vitamin D, health effects, and sun exposure, many people have formed opinions about vitamin D. However, no study has quantified these beliefs among Americans and examined whether they are associated with sun exposure and protection practices. The primary purpose of the current study was to explore beliefs about of vitamin D in the United States and identify demographic and behavioral characteristics associated with these perceptions. Specifically, this study sought to determine if perceptions about vitamin D are associated with sun protective behaviors, exposure, and sunburns. This data might help us understand factors that may influence beliefs and may be a foundation for vitamin D and sun protection education.

## 2. Method

This study used data from a cross-sectional survey of three groups of adults (e.g., lifeguards, pool managers, and parents). Data were collected in 2006 from participants in a large randomized controlled trial of a pool-based, sun protection program called “Pool Cool” [21,22]. The program and research were conducted in hundreds of locations in the United States [22]. 

### 2.1. Participants and Procedures

A total of 3,922 adults from 262 swimming pools across the United States responded to a survey at the end of the summer of 2006 (August–October). Participants had completed a beginning-of-summer survey on-site at the swimming pool and were re-contacted either at their respective pools (lifeguards and pool managers) or over the phone by a contracted telephone researcher and polling agency (parents) to fill out a sun related questionnaire. The overall response rate among respondents who had completed the first survey was 54.4% (3,922/7,208), with those younger (*p* < 0.001), married (*p* < 0.01), and Caucasian (*p* < 0.001) more likely to participate in the second survey. 

### 2.2. Measures

Measures were from surveys on skin cancer and sun protection previously used in literature [21,23,24,25,26]. Participants were asked to report how much they agreed with three vitamin D related belief statements. The questions read: “How much do you agree with the following?” (1) People need to go out in the sun to have enough vitamin D to be healthy; (2) You can get enough vitamin D from foods such as fortified milk and orange juice; and (3) Sunlight helps the body to naturally produce vitamin D. Response options were based on a 5-point Likert scale that ranged from 1 (strongly disagree) to 5 (strongly agree). 

Sun protection, sun exposure and sunburn questions were standard items used in previous studies [22,27]. Sun protective behaviors were assessed in regards to how often they practiced each five different behaviors (e.g., use of sunscreen, wearing a hat, wearing a shirt, seeking shade, and wearing sunglasses) on a 5-point scale from 1 (strongly disagree) to 5 (strongly agree). Measures of regular sun exposure were asked in average hours per day between 10 a.m. and 4 p.m. on weekdays and weekends. Sunburn was assessed by asking “How many times this summer did you get a sunburn?” 

### 2.3. Data Analysis

The primary aim of this study was to describe beliefs in response to questions about vitamin D and to identify significant demographic predictors and behavioral and sunburn correlates of vitamin D perceptions. All data analyses were performed using PASW software (Chicago, IL, USA) version 18 [28]. Preliminary descriptive statistics were calculated to describe the distribution of age, gender, ethnicity, education, number of hours of sun exposure per day, average number of sun protective behaviors, and sunburns. Ordinal polytomous universal model regressions (PLUM) were used to determine the associations between demographic characteristics and the vitamin D questions for each participant group. PLUM regressions were chosen to account for the ordinal nature of the outcomes and provide standard odds ratio estimates and significance tests. Linear regression models were used to assess the effects of each vitamin D question to the sunburn, sun protection, and sun exposure variables. 

## 3. Results

Table 1 describes background characteristics for lifeguards, pool managers, and parents. A total of 262 swimming pools were registered to participate during the summer of 2006, representing 27 separate metropolitan regions across the United States. Surveys were returned from 245 of these pools (93.5%) with a total of 3,922 participants (1,940 lifeguards, and 162 pool managers, and 1,820 parents). The majority of respondents were Caucasian (82%) and female (72%). Of these pools, 50% were located in communities of less than 100,000 people. Average weekly attendance of the pools ranged widely with 52% admitting fewer than 1,000 people and 21% serving more than 2,000 people per week.

**Table 1:** Characteristics of Participants (N = 3,922), 2006, USA.

	Lifeguard ( *n* = 1,940)	Pool Manager ( *n* = 162)	Parent ( *n* = 1,820)
Mean Age (SD)	18.8 (5.35)	29.3 (10.94)	37.7 (9.36)
Sex (%)			
Male	40.1	38.3	12.4
Female	59.8	61.7	85.9
Race (%)			
Caucasian	86	88.9	77
Non-Caucasian	14	11.1	23
Marital status			
Married	2.7	26.5	86.9
Non-married	95.7	72.8	12.4
Education (%)			
High school or less	66.7	0	1.3
Some college of more	32.4	50.6	42.2
College degree or more	0.9	49.4	56.5
Sun related behaviors			
Sun exposure, hours per day (SD) *	4.52 (1.31)	3.87 (1.58)	2.12 (1.09)
Sun protection habits			
score (SD) *	2.60 (0.59)	2.73 (0.56)	2.93 (0.76)
# of sunburns during			
summer season(SD) *	2.44 (1.32)	2.01 (1.11)	1.31 (0.66)

Note: * *p* < 0.05

The frequency of sunburns, and amount of sun exposure and sun protection practices differed significantly across the three participant groups. Lifeguards had the highest average number of sunburns, reported the most average sun exposure, and participated in the fewest sun protection behaviors. Conversely, parents reported the most sun protective behaviors and fewer hours of sun exposure. 

In regards to the three vitamin D perception items, Table 2 summarizes the responses the vitamin D belief questions. Overall, the pattern of agree/disagreeing were quite similar, but approximately half of the parents agreed with all the statements.

**Table 2:** Distribution of Responses to the Three Statements about Vitamin D.

	Strongly Disagree	Disagree	Neutral	Agree	Strongly Agree
	%	%	%	%	%
People need to go out in the sun to have enough vitamin D to be healthy					
Lifeguard	5.2	21.3	36.8	31.1	5.7
Pool Manager	8.6	32.7	31.5	22.8	4.3
Parent	5.7	32	17.5	40.1	4.7
You can get enough vitamin D from foods such as fortified milk and orange juice					
Lifeguard	1.5	13.4	37.3	39.7	8.1
Pool Manager	3.7	13.6	35.8	37	9.9
Parent	1.7	25.2	19.2	48.3	5.7
Sunlight helps the body to naturally produce vitamin D					
Lifeguard	1	4.5	38.8	45.3	10.4
Pool Manager	3.1	7.4	40.1	41.4	8
Parent	1.3	9.8	19.8	58.5	10.7

### 3.1. Demographic Correlates of Vitamin D Beliefs 

Table 3 shows results comparing levels of endorsement of vitamin D items predicted by demographic characteristics. Results indicated that lifeguards and pool managers who were non-Caucasian were less likely to agree with the statement that “People need to go out in the sun to have enough vitamin D to be healthy.” Interestingly, female lifeguards were more likely, while female pool managers were less likely than their male counterparts to agree with this belief. Second, for “You can get enough vitamin D from foods such as fortified milk,” the only statistically significant result was that female parents were more likely than male parents to agree with this as being true (*OR* = 1.45, *CI:* 1.11–1.90; *p* < 0.001). Lastly, lifeguards and parents who were non-Caucasian and those with lower education levels were less likely to agree the statement, “Sunlight helps the body to naturally produce vitamin D.” 

**Table 3:** Multivariate Ordinal Regression for Beliefs by Demographic Groups.

	Need sun (D1) ^^^	Food as source (D2) ^^^	Sunlight helps production (D3) ^^^
	OR	OR	OR
**Lifeguard**			
*Education*			
High school or less	1.09 (0.92-1.30)	1.07 (0.90-1.27)	0.72 *** (0.60-0.86)
4 year degree or more (referent)			
*Ethnicity*			
Non-White	0.75 * (0.59-0.95)	1.09 (0.85-1.38)	0.70** (0.55-0.90)
White (referent)			
*Sex*			
Female	1.24 * (1.05-1.47)	0.97 (0.82-1.15)	1.17 (0.99-1.39)
Male (referent)			
**Pool Manager**			
*Education*			
Some college or less	0.82 (0.47-0.143)	1.06 (0.61-1.86)	1.09 (0.61-1.92)
4 year degree or more (referent)			
*Ethnicity*			
Non-White	0.41 * (0.17-0.97)	2.37 (0.99-5.70)	0.80 (0.33-1.91)
White (referent)			
*Sex*			
Female	0.55 * (0.31-0.97)	1.33 (0.75-2.38)	0.74 (0.41-1.34)
Male (referent)			
**Parent**			
*Education*			
Some college or less	0.89 (0.75-1.06)	0.98 (0.82-1.17)	0.62 *** (0.52-0.75)
4 year degree or more (referent)			
*Ethnicity*			
Non-White	0.98 (0.79-1.20)	1.14 (0.92-1.42)	0.73 ** (0.59-0.91)
White (referent)			
*Sex*			
Female	0.87 (0.67-1.13)	1.45 ** (1.11-1.90)	0.97 (0.73-1.28)
Male (referent)			

Note: * *p* < 0.05, ** *p* < 0.01, *** *p* < 0.001; ^^ ^Vitamin D1 = People need to go out in the sun to have enough vitamin D to be healthy; ^^ ^Vitamin D2 = You can get enough vitamin D from foods such as fortified milk and orange juice; ^^ ^Vitamin D3 = Sunlight helps the body to naturally produce vitamin D.

### 3.2. Vitamin D Beliefs and Sunburns, Sun Exposure and Sun Protection

Results indicated that a stronger belief for the statement, “People need to go out in the sun to have enough vitamin D to be healthy,” predicted longer sun exposure (*p* = 0.001) for lifeguards. For parents, a stronger belief for the statement, “You can get enough vitamin D from foods such as fortified milk and orange juice,” predicted higher sun protection (*p* = 0.02), and a stronger belief for the statement, “Sunlight helps the body to naturally produce vitamin D,” predicted lower sun exposure (*p* = 0.04). There were no significant beliefs that predicted any sun related behaviors for pool managers.

## 4. Discussion

This study examined vitamin D related beliefs and sun exposure and protection behaviors among a broad US sample of lifeguards, pool managers, and parents. Overall, it was found that less educated, non-Caucasian lifeguards had lower perceptions about using ultraviolet sources as a vitamin D source while significantly staying longer in the sun and practicing lower sun protection behaviors than pool managers or parents. The differences between perceptions and actual behaviors (e.g., not using sunscreen) may be due to the fact that most lifeguards are highly sun exposed and practice poor sun safety habits [25,29,30]. In addition, the lifeguards were mainly in their late teens and early twenties, an age group that typically reports poor sun safety habits [29]. This study also supports previous studies showing that non-Caucasians report less sun protective behaviors than Caucasians [12,31]. Vitamin D deficiency is very common among minority groups but knowledge about vitamin D and sun protective behaviors among minorities is still limited [12]. In future studies, it will be important to conduct more vitamin D knowledge and sun protective behavior studies among minorities especially to reduce racial disparities in health [32,33].

Parents reported the highest average of sun protective behaviors and lowest average of sun exposure. An interesting notion was that parents who had a stronger belief in sunlight helping the body to produce vitamin D lead to lower sun exposure. This might be due to the fact that parents also had strong beliefs that they can obtain enough vitamin D from alternate sources such as fortified foods, which in turn might have lead them to lower sun exposure. Although this study did not examine parents’ vitamin D beliefs and sun protective behaviors towards their children, a recent study examining attitudes and behaviors related to sun protection and vitamin D in Australia showed that over 77% of adults thought that their child was maintaining a healthy level of vitamin D. However, only 12% were actually concerned about their maintaining a healthy vitamin D level, and nearly 14% had actually changed the sun protection behaviors for their child [34]. It might be interesting in a future study to compare the same parent beliefs and how it effects their sun protective behaviors towards their children in America. 

Overall, results from this study show differences in vitamin D beliefs and sun protective behaviors among lifeguards, pool managers, and parents. With the current lack of clarity about vitamin D beliefs and sun protective behaviors [35,36,37,38], it is important for future studies to test educational programs about the importance of vitamin D and sun protective behaviors. A possible strategy might be to create specific narrative messages. Previous research has suggested that specific narrative messages may be a powerful means for promoting positive health actions [22]. One study examined the effectiveness of a targeted, peer driven skin cancer prevention program for lifeguards, also known as Pool Cool Plus [29]. Results showed that the targeted program was effective in decreasing sunburn and improving sun protection behaviors and pool policies. Similar to the Pool Cool Plus study [29], future research should expand upon sun protective educational programs by creating narrative tailored educational programs across different demographic populations (e.g., non-Caucasian lifeguards). By creating narrative tailored programs across certain groups, it might be an effective way to promote vitamin D knowledge and promote positive health actions [39,40].

## 5. Limitations

While this study had a large sample, the majority were Caucasian and female and therefore, results may not be not generalizable to other groups. Second, the three belief items do not represent the full range of possible beliefs about vitamin D that are relevant to sun safety and health. Also, the findings are based on a single cross-sectional survey of adults who were exposed to a sun safety program, though the program did not include specific content about vitamin D and its effects and sources. Lastly, data was collected in 2006 which might not reflect current vitamin D trends. However, with the current inconsistencies of vitamin D knowledge, this study adds important information about public opinions on vitamin D knowledge and sun safety behaviors in America.

## 6. Conclusions

This study provides new information regarding perceptions and beliefs about vitamin D knowledge among lifeguards, pool managers, and parents around the nation. This study also allows us to predict certain vitamin D perceptions and sun related behaviors across different demographic characteristics. Having proper knowledge is vital in making conscious health decisions [19]. With current data reporting that sun related knowledge and behaviors differ across certain demographic characteristics, future research might want to expand upon previous sun protection educational programs by creating specific vitamin D educational programs that can target certain groups according to beliefs about vitamin D and demographic characteristics. By increasing vitamin D knowledge, the main goal will be to increase healthy vitamin D intake while sustaining sun safety behaviors to further improve sun safety behaviors across the nation.
